# Obesity, Metabolic Syndrome, Diabetes and Kidney Stones: Strengthening Links Over Time?

**DOI:** 10.17925/EE.2026.22.1.7

**Published:** 2026-03-16

**Authors:** Ides M Colin, Agnieszka Pozdzik

**Affiliations:** 1. REMEDIAB Center, CHU HELORA, Mons, Belgium; 2. Nephrolithiasis Clinic, CHU HELORA, Mons, Belgium

**Keywords:** Cardiometabolic syndrome, diabetes mellitus, type 2, GLP-1 receptor agonists, nephrolithiasis, obesity, pharmacological mechanisms of action, sodium-glucose transporter 2 inhibitors

## Abstract

Long considered separate entities, obesity/type 2 diabetes/metabolic syndrome and kidney stone disease are now recognized as interconnected conditions, both from an epidemiological and pathophysiological perspective. Although the pathophysiological mechanisms between visceral obesity, metabolic syndrome and type 2 diabetes are well established, the link with an increased propensity to develop kidney stones may appear less clear at first glance. Over the past decade, data from retrospective analyses and prospective cohort studies have clarified the nature and the magnitude of this relationship. The common threads linking these pathophysiological entities, apparently distinct at first sight, are insulin resistance and systemic low-grade inflammation, which should be considered as the unifying pathological roots. Within the kidney, higher urinary levels of oxalate, calcium and uric acid, combined with a fall in urine pH, increase supersaturation of lithogenic salts. If unopposed, this physicochemical imbalance drives crystal nucleation, growth and aggregation, leading to precipitation of insoluble salts and, ultimately, to stone formation. This narrative review presents epidemiological and pathophysiological evidence that helps understand the extent to which seemingly unrelated conditions are, in fact, part of a broader metabolic disorder, the cardiovascular–kidney–metabolic syndrome, in which kidney stone disease can likely be integrated because common pathophysiological features are shared. The question then arises as to whether weight loss and modern treatments for type 2 diabetes, in particular glucagon-like peptide-1 receptor agonists and sodium–glucose cotransporter-2 inhibitors, influence the lithogenic risk.

Urinary lithogenesis processes are activated in patients with obesity, metabolic syndrome (MetS), and type 2 diabetes (T2D). This is a relatively unfamiliar topic among endocrinologists. Beyond the epidemiological evidence accumulated over the recent years, the purpose of this paper is to dive into compelling pathophysiological arguments that connect these different conditions.

## The pathophysiological links between obesity, metabolic syndrome and type 2 diabetes: a well-known paradigm

Obesity is a chronic, multifactorial disease, defined by an excessive and abnormal accumulation of fat, which results in an increased risk of adverse health outcomes, including metabolic syndrome, type 2 diabetes, hypertension, cardiovascular diseases (CVDs), chronic kidney disease (CKD), cancers, neurodegenerative diseases and various complications, including kidney stone disease (KSD).^1–9^

The accumulation of fat in the omental compartment triggers events that, from a pathophysiological perspective, link obesity with MetS and T2D. As visceral fat accumulates, adipocytes become hypertrophied (versus hyperplastic in the subcutaneous fat compartment), and their capacity to normally store fat is rapidly exceeded. This leads to a state of chronic, low-grade systemic inflammation, which plays a central role in the development of insulin resistance. Insulin resistance results in the release of increased amounts of free fatty acids directly into the portal vein and surrounding organs, further exacerbating the state of systemic insulin resistance.^10^

The combined action of free fatty acids and pro-inflammatory cytokines disrupts insulin signalling pathways in insulin-sensitive tissues, particularly in skeletal muscle cells, where glucose uptake is impaired, and in the liver, where glucose production increases.^11^ Ultimately, this affects pancreatic beta cells, which, under the influence of metabolic stress, are no longer able to sustain insulin secretion that compensates for peripheral insulin resistance. The decline in insulin production eventually leads to T2D.^12^

## Can the links between obesity/metabolic syndrome/type 2 diabetes be extended to kidney stone disease?

### Epidemiological arguments

Over the last three decades, a rather striking synchronicity has been observed in the epidemiological increase in these different pathophysiological conditions. A global increase in the prevalence of overweight and obesity has been observed, with the percentage doubling in the adult population over the last 30 years.^8^ The prevalence of T2D follows the same trend and is estimated to have quadrupled over the same period.^13^ Recent data indicate that the incidence of KSD has also increased significantly over time, following a very similar pattern to the obesity/diabetes epidemics. The prevalence of KSD has doubled over the past 30 years, particularly among females of reproductive age.^14^ These concurrent epidemiologic increases are more likely due to multiple shared determinants, including convergent pathophysiologic pathways, rather than a single isolated trend, which supports the hypothesis of genuine epidemiologic and mechanistic links between these conditions.

The risk of developing KSD is estimated to be 1.36–1.5 times higher in people with obesity than in lean people, independent of other metabolic abnormalities.^15–17^ Studies have clearly reported that obesity is associated with an increased risk of KSD.^15,18,19^ In the prospective UK Biobank cohort (n=487,860 participants free from kidney stones at baseline; mean follow-up period of 12.6 years), multivariable Cox models showed that the risk of KSD significantly increased for each 5-unit increase in body mass index (BMI) (hazard ratio [HR]: 1.19; 95% confidence interval [CI]: 1.16–1.20), for each 10 cm increase in waist circumference (HR: 1.15; 95% CI: 1.10–1.20), for each 0.05-unit increase in waist-to-hip ratio (HR: 1.09; 95% CI: 1.07–1.11) and for each 5% increase in fat mass (HR: 1.16; 95% CI: 1.13–1.19).^20^

The risk of developing KSD is increased among individuals with MetS.^21^ According to a recent study, the prevalence of KSD triples among individuals who have five MetS traits.^22^ Two other meta-analyses also confirmed a clear association between MetS and KSD.^23,24^

The risk of KSD increases as the number of MetS traits increases. It is more than a simple correlation; rather, the accumulation of MetS traits represents an increased risk of developing KSD.^25^ Data from the largest prospective cohort, UK Biobank, confirm the close relationship between the number of MetS traits and KSD.^20^ Compared with no traits, the adjusted HRs (95% CI) were 1.19 (1.09–1.29) for one trait, 1.38 (1.25–1.52) for two traits, 1.47 (1.32–1.65) for three traits, 1.59 (1.39–1.82) for four traits and 1.82 (1.43–2.33) for five traits.^20^ The pathophysiological link that connects these different conditions is insulin resistance, which plays a key role.^26^

This synchronicity also exists when T2D is considered, given its strong association with both metabolic dysregulation and kidney stone formation.^27^ T2D was independently associated with incident stones (adjusted HR: 1.14; 95% CI: 1.04–1.21) in the Cox framework of UK Biobank participants.^20^

**Figure 1:**
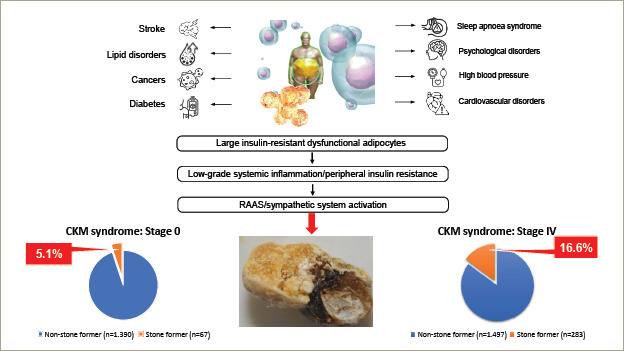
Links between visceral obesity/metabolic syndrome/type 2 diabetes and kidney stone disease

More recently, the concept of cardiovascular–kidney–metabolic (CKM) syndrome has been proposed by the American Heart Association (AHA).^28,29^ It is a health disorder resulting from connections between obesity, diabetes, CKD and CVD. According to the AHA, CKM stages range from: stage 0 (no CKM risk factors), stage I (excess/dysfunctional adiposity), stage II (metabolic risk and/or moderate–high CKD risk), stage III (subclinical CVD with CKM risk, very high-risk CKD or high predicted CVD risk) and stage IV (clinical CVD with CKM risk; IVa without kidney failure, IVb with kidney failure).^28,29^ Recently, the National Health and Nutrition Examination Survey data (2007–2020; n=15,568) showed that self-reported stone prevalence rose stepwise with advancing CKM stage – from 5.10% at stage 0 to 16.55% at stage IV (p<0.001). Fully adjusted odds ratios versus stage 0 were 1.72 (95% CI: 1.28–2.32) at stage II, 2.00 (1.29–3.10) at stage III and 2.36 (1.64–3.40) at stage IV.^30^ Collectively, these findings support conceptualizing KSD within the cardio–kidney–metabolic continuum, favouring integrated, risk-directed management rather than an exclusive urological approach (*Figure 1*).

*Visceral adiposity drives lipotoxicity and inflammation, promoting insulin resistance, dyslipidaemia and hyperglycaemia; failure of pancreatic compensation leads to T2D. RAAS/sympathetic activation contributes to hypertension and cardio–metabolic–kidney damage. Nephrolithiasis prevalence increases across CKM stages (≈5.1–16.6%). This dysmetabolic milieu alters urine composition, increasing lithogenic risk and potentially causing crystal-related tubular injury. Figure was created using Microsoft Powerpoint*

*CKM = cardiovascular–kidney–metabolic; RAAS = renin–angiotensin–aldosterone system; T2D = type 2 diabetes.*

## Pathophysiological arguments

### Insulin resistance as a lithogenic switch: an acid–base framework (net acid excretion, ammonium ion and urine pH)

In insulin resistance and related dysmetabolic states, impaired proximal tubular ammoniagenesis reduces the kidney’s capacity to excrete acid as ammonium ion (NH_4_^+^), the principal buffered component of net acid excretion (NAE; NAE ≈ NH_4_^+^ + titratable acidity - urinary bicarbonate). When endogenous acid generation is sustained or increased, reduced NH_4_^+^ buffering increases reliance on titratable buffers, which are capacity-limited and typically lower urinary pH to maintain proton excretion.^31,32^ This framework provides a mechanistic basis for the reported association between insulin resistance, a persistently low-urine-pH phenotype and an enhanced risk of uric acid (UA) crystallization.

### Uric acid nephrolithiasis in dysmetabolic states: low urine pH as the dominant driver

Although insulin resistance is an intuitively plausible pathophysiologic bridge linking obesity, MetS and T2D to KSD, its causal relevance has been interrogated.^33,34^ Nevertheless, substantial mechanistic evidence supports a true link, largely derived from studies of UA nephrolithiasis in insulin-resistant states.^35^ Of note, UA stones in this context are not primarily explained by increased urinary UA excretion.^35^ Rather, the dominant abnormality is acidic urine. Under comparable dietary and fluid conditions, UA stone formers exhibit lower urinary pH (e.g. ~5.7 in UA stone formers versus ~6.17 in controls).^36^ These observations support the concept that low urinary pH, rather than hyperuricosuria, determines UA crystallization in a state of insulin resistance. Consistently, Sakhaee et al. showed that UA stone formers have impaired urinary buffering capacity across a range of dietary acid loads, involving reduced NH_4_^+^ excretion as a central defect.^37^

Biological plausibility is reinforced by renal tubular physiology.^31,32^ Insulin receptors are expressed along the tubular epithelium, and insulin normally facilitates renal acid handling by promoting NH_4_^+^ buffering and stimulating key transport processes, including proximal tubular sodium/hydrogen exchanger-3 (NHE3)-mediated sodium ion (Na^+^)/NH_4_^+^-dependent exchange. In insulin-sensitive individuals, insulin administration increases urinary pH and NH_4_^+^ excretion.^33^ At the cellular level, this effect can be explained by enhanced NH_4_^+^ secretion across the apical membrane of proximal tubular cells via the NHE3-mediated Na^+^/NH_4_^+^ exchanger, with additional contribution from parallel H^+^ and ammonia (NH_3_) transport.^32^ In insulin resistance, renal ammoniagenesis and/or NH_4_^+^ excretion is reduced. Thus, secreted acid is less effectively buffered, and urinary pH remains chronically low, an environment that strongly favours UA precipitation.^36^ This model is reinforced by population data showing that urinary pH decreases as the number of MetS traits increases, even in individuals without stones, suggesting that insulin resistance generates a ‘lithogenic acidic urine phenotype’ upstream of overt clinical stone disease.^38,39^

### Diabetes is not sufficient: phenotype susceptibility and impaired ammonium ion response to acid load

It is noteworthy that T2D alone is not sufficient to cause UA stones, as most patients with diabetes never form them. Comparative physiologic studies suggest that UA stone formers carry an additional susceptibility beyond insulin resistance-associated urine acidification. In a prospective BMI-matched study, Bobulescu et al. compared non-diabetic UA stone formers, diabetic non-stone formers and non-diabetic non-stone-forming controls.^40^ Although both UA stone formers and diabetic non-stone formers had lower urinary pH than controls, only UA stone formers showed an impaired NH_4_^+^ excretory response to an acute acid load, indicating a superimposed defect in ammonium buffering capacity that may allow sorting out individuals who crystallize UA from those with ‘acidic urine’ alone.^40^ More broadly, the low urinary pH observed in T2D appears to reflect a dual mechanism: higher NAE together with reduced availability of NH_3_ buffers.^41^

Under controlled metabolic-diet conditions across three groups (74 UA stone formers, 13 patients with T2D without stones and 51 healthy volunteers), both UA stone formers and diabetic participants exhibited higher NAE, lower urinary pH and a lower fraction of NAE excreted as NH_4_^+^ (NH_4_^+^/NAE) than healthy controls. Critically, UA stone formers had an even lower NH_4_^+^/NAE than diabetics without stones. Moreover, although higher total/truncal fat correlated with lower NH_4_^+^/NAE in healthy volunteers, this relationship was lost in the diabetes and UA stone-former groups, because NH_4_^+^/NAE remained persistently low across a wide range of adiposity, supporting a partly diet-independent dual defect (increased acid production plus impaired renal ammonium excretion) as a key mechanism of UA stone pathogenesis.^42^

### From urine pH to crystals: pH-dependent uric acid solubility and the pKa threshold

A persistently low urinary pH promotes UA nucleation, the first step in crystallization, which may progress to aggregation and stone formation. The solubility of UA is strongly pH-dependent and decreases in an acidic environment. UA tends to precipitate when urinary pH falls below its pKa (≈5.5), favouring the poorly soluble, undissociated form.^43^ Conversely, when urinary pH rises above ~5.5, UA is converted into its ionized, water-soluble urate form (A^-^), which is less prone to crystallization. Maintaining urinary pH above this threshold is therefore essential for both the prevention and management of UA stones. This mechanism helps explain the increased incidence of UA stones in patients with T2D or insulin resistance, even with normal or only moderately elevated urinary UA excretion.^35,43^

### Beyond uric acid: dysmetabolism and calcium oxalate stones (oxalate, calcium, citrate)

Collectively, these physiologic and physicochemical data support a coherent model in which insulin resistance promotes UA lithogenesis primarily through persistently low urine pH, driven by altered partitioning of NAE (reduced NH_4_^+^ buffering relative to acid load). The dysmetabolic milieu also promotes calcium stone risk through distinct pathways.

The formation of calcium oxalate (CaOx) stones – the most common type of kidney stones (~80%) – is also promoted. Obesity itself stimulates urinary oxalate excretion, originating from both dietary sources and endogenous production.^2,44^ Obesity-associated low-grade inflammation and dysmetabolism may increase intestinal oxalate absorption, thereby increasing free urinary oxalate available to complex with calcium and raising CaOx supersaturation.^45^ Concomitant hypercalciuria, frequently observed in obesity and MetS, further elevates the ionic activity product for CaOx, promoting nucleation and crystal growth. Increased urinary calcium availability is also exacerbated by higher sodium and animal protein intake.^46^ Accordingly, 24 h urinary calcium excretion correlates with urinary sodium excretion (*r*=0.38; p<0.001) and estimated dietary protein intake (*r*=0.44; p<0.001).^46^

Another mechanism involved in CaOx stone formation may originate from metabolic dysfunction-associated steatotic liver disease (MASLD). Recent studies suggest that hepatic oxalate production is increased in MASLD, supporting a link between endogenous oxalate generation, insulin resistance and KSD.^47^ Through downstream effects on urine composition, MASLD may therefore promote CaOx crystallization.^48^

MetS is also associated with hypocitraturia. A diet high in animal proteins, combined with excessive sodium intake, can further decrease urinary citrate excretion. Citrate is a major endogenous stone inhibitor: it chelates urinary calcium to form soluble calcium-citrate complexes, reducing free Ca²^+^ available to bind oxalate, thereby inhibiting CaOx crystal formation. Thus, hypocitraturia, common in obesity and MetS, removes this protection and is a major, modifiable driver of KSD.^49^

### Crystal–tubule injury as an amplifier: inflammation, oxidative stress and chronic kidney disease progression

In addition, increased release of pro-inflammatory cytokines (e.g. interleukin-6) and C-reactive protein may contribute to diabetic kidney disease.^50,51^ Kidney dysfunction resulting from diabetic glomerulopathy, obesity-associated tubulopathy and KSD can lead to hypertension, further exacerbating insulin resistance and endothelial dysfunction, and thereby perpetuating a potentially uncontrollable vicious cycle of organ damage. Therefore, the progression of the different pathological mechanisms is neither linear nor occurring in parallel, but rather in close interaction, influencing and strengthening each other, thus increasingly exacerbating the pathophysiological process.

This process is even more complex when realizing that kidney stones themselves can directly harm renal tubule cells through local inflammation, fibrosis and oxidative stress.^52^ Of course, crystal- and stone-related tubular injury is a global mechanism in nephrolithiasis and is not specific to obesity or T2D. Injured tubular epithelial cells can become nucleation sites for further crystal formation, which, in turn, triggers additional local inflammation, creating a self-perpetuating cycle. In particular, CaOx crystals that accumulate in the tubules induce mitochondrial dysfunction in tubular epithelial cells and contribute to reactive oxygen species generation, which is responsible for cell damage.^51^ The activation of apoptosis and of the Nucleotide-binding and Oligomerization Domain-like receptor family, pyrin domain containing three inflammasome, a multi-protein complex of the innate immune system involved in various inflammatory diseases, has also been implicated in crystal-induced tubular injury.^53,54^

### Papillary phenotypes and chronic kidney disease risk: Randall’s plaques versus ductal plugging

Vascular dysfunction occurring at the level of the *vasa recta* is more frequent in cases of diabetes and MetS. Because of this vascular dysfunction, the renal papilla, which is characterized by relative hypoxia, increased osmotic pressure and turbulent blood flow, may become a suitable site for the formation of Randall’s plaques. Calcification processes can be activated with hydroxyapatite deposition (the main component of Randall’s plaques), which can act as nuclei for crystal aggregation.^55^ The formation of Randall’s plaques is an interstitial process. Sub-urothelial apatite forms beneath ostensibly normal papillae; focal epithelial thinning exposes plaques, allowing CaOx accretion. Stone detachment avulses small, calcified islands to produce limited focal nephron loss. Immune profiling shows macrophage-predominant cuffs and low-grade spatially constrained inflammation. As revealed by high-resolution endoscopy and papillary biopsy, another mechanism may explain CaOx nephrolithiasis. Large mineral casts form within terminal collecting (Bellini) ducts, obstructing flow and disrupting tubular epithelium.^56,57^ Because each terminal duct drains many upstream nephrons, plugging can precipitate regional nephron dropout, papillary atrophy and secondary glomerular loss. Emerging data reveal diffuse T-lymphocyte enrichment with tubulitis, consistent with an adaptive immune response that may amplify crystal tubular toxicity and interstitial fibrosis. Clinically, phenotype matters: while plaque-dominant disease is often indolent, plugging-dominant disease plausibly carries a higher long-term risk of CKD progression.^58^

## Clinical implications: early metabolic screening of kidney stone disease in cardiovascular–kidney–metabolic phenotypes

**Figure 2:**
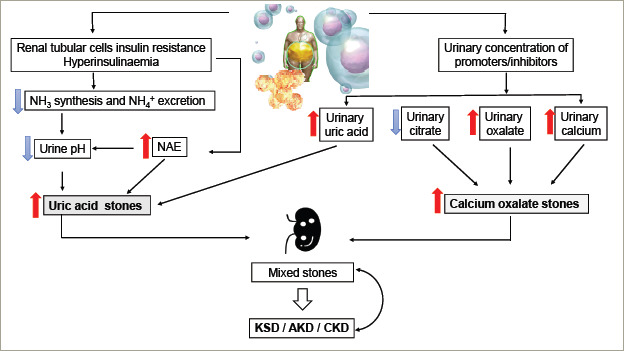
Lithogenic processes triggered by insulin resistance and low-grade inflammation

Taken together, obesity- and MetS-driven inflammation, coupled with local renal micro-inflammation, not only directly impairs kidney function but also promotes crystal nucleation, adhesion and growth. The resulting tubular epithelial cell injury perpetuates intratubular lithogenesis and amplifies interstitial inflammation, creating a self-reinforcing vicious cycle. KSD in people with obesity/MetS/diabetes may represent the visible tip of the complex dysmetabolic iceberg. We suggest that subtle alterations in urinary composition arise in the very early steps of KSD. Thus, although the proportion of patients with KSD at CKM stage IV may appear rather low (≈16.5%), urine composition abnormalities and microcrystal formation likely affect a much larger group of patients, yet remain clinically silent.^30^ If persistent urinary supersaturation is not corrected, crystal-induced structural injury of tubular cells and interstitial fibrosis may worsen over time. In this context of systemic low-grade inflammation and reduced renal reserve, regular urinary metabolic screening and targeted preventive strategies are essential to prevent stone formation and protect kidney function.^59^ Obviously, the increased prevalence of KDS associated with obesity and MetS is a complex process resulting from multiple factors, whose interactions combine to promote a lithogenic environment. Therefore, it is not a single factor that explains the lithogenic profile associated with obesity, but rather a complex interplay involving urinary pH, sodium, calcium, oxalate, UA and citrate levels, within a context of low-grade inflammation and insulin resistance (*Figure 2*).

### Is weight loss likely to influence the prevalence of kidney stone disease?

Sustained long-term weight loss is the cornerstone of the strategy to reduce the risk of nephrolithiasis in individuals with obesity/MetS/T2D.^60^ Weight loss, secondary to lifestyle measures combining dietary changes and increased physical activity, has a direct effect on abdominal obesity and helps to reduce insulin resistance.^61^ Beyond the evident impact on cardiovascular events and the development of T2D, weight reduction also has a favourable effect on the incidence of KSD.^61^ Among other metabolic benefits, favourable modifications in urinary composition occur, including decreased urinary calcium excretion and increased citrate excretion, thereby contributing to decreased risk of calcium stone formation. Moreover, improvement in insulin resistance enhances renal ammoniagenesis, elevates urinary pH and decreases the supersaturation, precipitation and crystallization of UA, thereby lowering the risk of UA stones.^61,62^

The amplitude of weight loss deserves special attention. Extreme diets and too rapid or too significant weight loss can paradoxically lead to increased urinary lithogenic factors.^61^ Thus, metabolic surgery, which is used as a treatment for severe obesity, is associated with a significantly increased risk (more than six times) of KSD. This increased risk is attributed to changes in intestinal absorption and fat malabsorption, leading to enteric hyperoxaluria.^63–67^ Bariatric surgery induces substantial and sustained weight loss (≈25–35% of baseline body weight at 1–2 years) but is consistently associated with a more lithogenic urinary profile, particularly after malabsorptive procedures.^68^ Post-surgery changes include increased urinary oxalate, reduced citrate, lower urine volume and higher CaOx supersaturation, translating into a 2–4-fold increased risk of kidney stone formation compared with non-surgical patients with obesity.^68^

*Insulin resistance and low-grade inflammation impair ammoniagenesis and lower urine pH, favouring UA stones. Reduced inhibitors (e.g. citrate) and increased promoters (e.g. oxalate, calcium) support calcium and mixed stones. Early crystals/plugs can injure tubules and amplify lithogenicity. Conversely, CKD further lowers pH/citrate and may increase oxalate, sustaining a vicious circle. See text for details. Figure was created using Microsoft Powerpoint*

*AKD = acute kidney disease; CKD = chronic kidney disease; KSD = kidney stone disease; NAE = net acid excretion; NH_3_ = ammonia; NH_4_^+^ = ammonium ion; UA = uric acid.*

When weight loss is driven by drugs such as orlistat or phentermine-topiramate, an increased risk of nephrolithiasis has been reported.^69,70^ Orlistat increases fat content in the intestine that binds calcium by saponification, resulting in increased intestinal oxalate permeability, thereby leading to increased urinary oxalate excretion (iatrogenic enteric hyperoxaluria).^69^ In susceptible patients, severe hyperoxaluria can cause intratubular CaOx precipitation and may sometimes progress to acute oxalate nephropathy (often presenting as acute kidney injury).^71–73^ Phentermine-topiramate increases calcium phosphate stone risk via the topiramate component, which inhibits carbonic anhydrase and produces metabolic acidosis with hypocitraturia and an alkaline urinary pH, thereby increasing calcium phosphate supersaturation and predisposing to calcium phosphate crystallization (including brushite).^74,75^ Population-based National Health and Nutrition Examination Survey (NHANES) analyses indicate an ~eightfold higher odds of recent stone events among current topiramate users.^76^

### What about sodium–glucose cotransporter-2 inhibitors and glucagon-like peptide-1 receptor agonists?

Regarding the treatment of T2D, the question arises about how sodium–glucose cotransporter-2 inhibitors (SGLT-2i) and glucagon-like peptide-1 receptor agonists (GLP-1 RAs) would influence urine composition and, consequently, the risk of KSD (*Table 1*).^77–86^

These two new drug classes are widely used in treating metabolic disorders and their consequences, based on the concept of CKM syndrome, as recently proposed by the AHA.^28,29^ Thus, beyond effects on glycaemic levels, it is now widely accepted that these molecules exert many favourable effects regarding cardiovascular and renal targets.^86,87^ Regarding the kidneys, the protective mechanisms are not yet fully understood, even though the beneficial effects likely involve local metabolic effects, particularly on micro-inflammation and the degree of insulin resistance, as well as favourable haemodynamic effects with a reduction, for example, in the intraglomerular vascular pressure.^86–89^ There are fewer data regarding the lithogenic profile associated with the use of these new drug classes. The question is, though, relevant since GLP-1 RAs have currently become one of the most effective drugs to treat obesity, with an incomparably more favourable safety profile compared with previously used agents.^90^

Across large observational studies, including a target trial in adults with recurrent nephrolithiasis and T2D using a Canadian general-population database, and meta-analyses, exposure to SGLT-2i is consistently associated with a lower risk of incident and/or recurrent nephrolithiasis compared with placebo or active comparators (e.g. GLP-1 RAs and dipeptidyl peptidase-4 inhibitors); a similar protective association has also been reported in nondiabetic populations.^77,80–82,84,91–95^

The mechanistic basis for this apparent benefit remains incompletely defined. At the level of urinary physicochemistry, SGLT-2i reproducibly increase urine volume via osmotic diuresis, thereby diluting lithogenic solutes and lowering supersaturation, a key determinant of crystal formation.^79^ Several studies suggest an increase in urinary citrate excretion, which may contribute to additional protection through calcium complexation and inhibition of crystal growth.^79,85,96^ In contrast, the effects on acid–base homeostasis and urine pH appear heterogeneous. Urine pH should be interpreted as an integrated output of NAE, ammonium buffering and net gastrointestinal alkali absorption (NGIA), rather than a direct readout of ‘ammoniagenesis’ alone.^77^

In the post-hoc Empagliflozin and Renal Oxygenation in Healthy Volunteers (EMPA-REIN; ClinicalTrials. gov identifier: NCT03093103) analysis conducted in healthy/overweight nondiabetic volunteers without kidney stones, empagliflozin lowered day- and night-time urine pH while increasing urinary citrate by ~50%; urinary NH_3_ decreased only numerically and did not reach statistical significance, so reduced ammoniagenesis remains hypothesis-generating in this setting.^79^

In Impact of the SGLT2 Inhibitor Empagliflozin on Urinary Supersaturations in Kidney Stone Formers (SWEETSTONE; ClinicalTrials. gov identifier: NCT04911660), a double-blind, placebo-controlled crossover phase II trial in nondiabetic stone formers, empagliflozin 25 mg/day reduced lithogenic surrogates, lowering relative supersaturation for calcium phosphate by 36% in calcium-stone formers and relative UA supersaturation by 30% in uric-acid stone formers versus placebo.^97^ Post-hoc acid–base analyses indicated that empagliflozin increased NGIA in both phenotypes, whereas NAE diverged – increasing in calcium-stone formers (+26%) and decreasing in uric-acid stone formers (-9%), a pattern that plausibly contributes to the opposite urine pH shifts observed between phenotypes (slightly lower versus placebo in calcium-stone formers and higher versus placebo in uric-acid stone formers).^77^

**Table 1:** Evidence summary of sodium–glucose cotransporter-2 inhibitors, urinary lithogenic profile (24 h urine and supersaturation) and nephrolithiasis outcomes^77–85^

Citation (first author; year)	Comparator	Study type	Population (n)	Urinary endpoint(s)	Main data/key results	Likely stone type impacted (UA versus CaOx)	Direction on pH/citrate/volume (reported)	Confidence tag (stone-type inference)
**Harmacek et al.; *JASN*, 2022** ^79^	Placebo	Post hoc analysis of randomized placebo-controlled trial (EMPA-REIN)	45 enrolled; 40 completed (empagliflozin 27; placebo 13); healthy volunteers	Split 24 h urine; pH, citrate; RSR CaOx/CaP/UA	pH ↓, citrate ↑; RSR CaP ↓; RSR CaOx ~ unchanged; UA supersaturation may ↑	CaP benefit likely; UA risk potentially ↑; CaOx uncertain	pH ↓; citrate ↑; volume ↔	Moderate (RSR measured, but not stone formers/no composition)
**Anderegg et al.; *Nature Medicine*, 2025^77^**	Placebo	Double-blind, placebo-controlled crossover phase II RCT (SWEETSTONE)	53 nondiabetic stone formers (28 calcium; 25 UA)	RSR CaOx/CaP/UA + 24 h urine chemistry	Calcium-stone group: RSR CaP ↓; UA-stone group: RSR UA ↓	CaP ↓ in calcium-stone formers; UA ↓ in UA-stone formers; CaOx not clearly ↓	Reported in trial: citrate ↑; pH direction differs by subgroup; volume ↔	High (stone type predefined + stratified + RSR endpoints by type)
**Schaub et al.; *Kidney 360*, 2025^85^**	Matched controls; also GLP-1 RA comparison	Retrospective cohort (cross-sectional + longitudinal subset)	Cross-sectional: SGLT-2i 124 (+620 controls). Longitudinal: SGLT-2i 59 pre/post	Volume, citrate, pH, UA, sulphate; SS CaOx/CaP/UA	Cross-sectional: volume ↑, citrate ↑, pH ↓; CaP SS improved; longitudinal signals weaker	Likely CaP benefit; UA mixed/uncertain; CaOx uncertain	Cross-sectional: pH ↓; citrate ↑; volume ↑ (longitudinal: NS after correction)	Moderate (SS/urine markers measured, but stone composition not measured)
**Bletsa et al.; *JCEM*, 2021^78^**	Other therapy/controls	Mechanistic metabolomics	T2D (dapagliflozin arm + comparators)	Urine metabolomics (1H-NMR)	Metabolomic shifts (not designed for stones/SS)	Cannot infer	Not reported for stone outcomes	Low
**Paik et al.; *JAMA*, 2024^83^**	GLP-1 RA; DPP-4i	Active-comparator new-user cohort (claims)	Very large matched cohorts	Not assessed	Lower nephrolithiasis risk with SGLT-2i versus comparators	Unknown subtype (mixed)	Not assessed	Low
**Kristensen et al.; *Diabetologia*, 2021^81^**	GLP-1 RA	New-user cohort (Danish registries)	12,325 matched pairs	Not assessed	Lower incident and recurrent nephrolithiasis with SGLT-2i	Unknown subtype (mixed)	Not assessed	Low
**McCormick et al.; *BMJ*, 2024^82^**	GLP-1 RA (± DPP-4i analyses)	Target trial emulation	T2D with prior nephrolithiasis	Not assessed	Lower recurrence with SGLT-2i versus comparators	Unknown subtype (mixed)	Not assessed	Low
**Shin et al.; *Diabetes Care*, 2025^84^**	DPP-4i	Target trial emulation (database)	Large PS-matched cohorts	Not assessed	Lower nephrolithiasis risk with SGLT-2i	Unknown subtype (mixed)	Not assessed	Low
**Kanbay et al.; *NDT*, 2024^80^**	Placebo/active (varied)	Systematic review + meta-analysis	Millions (pooled)	Not assessed	Pooled association suggests reduced risk	Unknown subtype (mixed)	Not assessed	Low

*‘Crystalluria outcome’ is marked ‘Not assessed’ when urine microscopy/crystal outcomes were not reported in the publication. Dapagliflozin, an SGLT-2i, was patented for the management of nephrolithiasis in 2009 (WO2009143021A1).*

*CaOx = calcium oxalate; CaP = calcium phosphate; DPP-4i = dipeptidyl peptidase-4 inhibitor; EMPA-REIN = Empagliflozin and Renal Oxygenation in Healthy Volunteers; GLP-1 RA = glucagon-like peptide-1 receptor agonist; H-NMR = proton nuclear magnetic resonance; NS = not significant; PS = propensity score; RCT = randomized controlled trial; RSR = relative supersaturation ratio; SGLT-2i = sodium–glucose cotransporter 2 inhibitors; SS = (urinary) supersaturation; SWEETSTONE = randomized, double-blind, placebo-controlled cross-over trial assessing the impact of empagliflozin on urinary supersaturations in kidney stone formers; T2D = type 2 diabetes; UA = uric acid.*

Among diabetic stone formers, cross-sectional comparisons have linked SGLT-2i use with higher urine volume and citrate, but lower urine pH; however, attenuation after adjustment in longitudinal analyses suggests that real-world pH differences may be partially confounded and should not be over-interpreted as a direct drug effect on ammoniagenesis.^96^ The effects of SGLT-2i on urine chemistry should therefore be interpreted – and, when possible, anticipated – in the context of marked heterogeneity in urinary lithogenic patterns. This variability reflects a constellation of factors, including patient phenotype, dietary habits and supplement use (e.g. high-dose vitamin C or other products that may increase oxalate burden), and comorbidities such as inflammatory bowel disease. Accordingly, current guidelines recommend that personalized stone prevention integrate targeted medical and nutritional interventions, guided by metabolic evaluation and stone composition analysis, and refined according to key patient-specific characteristics.^98,99^

Dapagliflozin (3 months) changed the urine metabolomic profile in patients with T2D independently of glucose lowering (no similar change was observed with insulin degludec). It increased urinary ketone bodies and several metabolites, including citrate, suggesting potential renal-beneficial metabolic effects that may contribute to renoprotection.^78^

Taken together, and in line with recent narrative syntheses, the current evidence supports a ‘trade-off’ model in which SGLT-2i are consistently favourable for calcium-salt risk (↑urine volume, ↑citrate, ↓calcium phosphate supersaturation), whereas effects on UA risk are context-and phenotype-dependent, driven largely by the direction and magnitude of urine pH change across populations and stone types.^100^ Overall, SGLT-2i appear to reduce lithogenesis primarily by lowering urinary supersaturation through increased urine volume and phenotype-specific acid–base modulation, rather than via a uniform alkalinizing or hypocalciuric mechanism. However, the cellular pathophysiological pathways potentially linking SGLT-2 inhibition to reduced crystal–tubular interactions (e.g. effects on tubular energetics, oxidative stress, inflammation or autophagy) remain insufficiently established in humans and should be considered exploratory.

SGLT-2i also exert local anti-inflammatory, as well as anti-fibrotic effects that contribute to reducing the expression of inflammatory markers, the expression of stone core matrix proteins and the adhesion and formation of CaOx crystals.^101^

These positive effects occur only under optimal conditions in the absence of episodes of prolonged osmotic diuresis potentially associated with an increased risk of dehydration. Conversely, in susceptible individuals, the combination of glycosuria and (when present) higher urinary pH may promote bacterial growth and increase the risk of urinary tract infection (UTI), although reported UTI signals are heterogeneous and appear to vary depending on the individual agent, sex and duration of exposure.^102^ Struvite stones may therefore arise from urinary infection with urease-producing micro-organisms (e.g. *Proteus*, *Klebsiella*, some *Staphylococcus* spp.). Urease hydrolyses urea into NH_3_ and carbon dioxide. NH_3_ generates NH_4_^+^ and HCO_3_^-^, further alkalinizing the urine. The resulting high pH promotes precipitation of struvite (magnesium ammonium phosphate hexahydrate [MgNH_4_PO_4_·6H_2_O]), typically admixed with carbonate apatite. These crystals often grow rapidly and can form staghorn calculi unless the infection is eradicated and urine is re-acidified.^103^

Regarding GLP-1 RAs, the data are less clear and much more controversial. A recent study using a large-scale systematic examination of 175 health outcomes from the US Department of Veterans Affairs suggested that GLP-1 RAs are associated with a higher rate of KSD compared with other antidiabetic medications.^104,105^ In contrast to weight-loss strategies with a recognized lithogenic risk (malabsorptive bariatric surgery, orlistat or topiramate), weight loss achieved with GLP-1 RA-based therapies in obese kidney stone formers was associated with a mean loss of 26.6 ± 7.3 kg and a significant reduction in 24 h urinary oxalate (40 ± 16 to 32 ± 11 mg/day; p=0.002), along with lower markers of dietary acid load – sulphate (21 ± 10 to 17 ± 9 mmol/day; p=0.005) and NH_4_^+^ (35 ± 22 to 29 ± 15 mEq/day; p=0.014) – without significant changes in CaOx, calcium phosphate or UA supersaturation, thereby suggesting an overall neutral-to-favourable lithogenic profile.^85,106^ The use of GLP-1 RAs has beneficial effects, of course, on renal health through systemic effects of lowering blood sugar, weight loss and certainly local effects on microinflammation.^107,108^ These systemic effects can contribute to a reduction in the risk of stones. It is also possible that they may have a negative effect on stone formation due to gastrointestinal adverse events, such as diarrhoea and dehydration; with obvious consequences on urine concentration and precipitation of solutes may occur. Thus, regarding GLP-1 RAs, additional studies are definitively required, inferring that a frank and clear opinion cannot yet be truly given. However, given their extremely beneficial effect in CKM, their use is obviously encouraged.

## Conclusions

In this article, we reported that conditions not traditionally viewed as directly related, such as obesity, MetS and T2D, as well as CKM, are, in fact, closely linked to KSD through both epidemiological associations and shared pathophysiological mechanisms. They are manifestations of a much broader metabolic disorder whose starting point is the accumulation of visceral fat, causing insulin resistance and chronic low-grade systemic inflammation. This biochemical environment creates a urinary environment conducive to KSD. This pro-lithogenic state is characterized by increased urinary excretion of calcium, UA and oxalate, as well as decreased citrate levels and altered urinary pH. It is therefore important for endocrinologists to always keep in mind, beyond renal disease as a complication of diabetes, the predisposition of patients with dysmetabolic conditions to develop kidney stones. The vicious circle that gathers these different pathological conditions by feeding on each other can be stopped and even reversed through a multidisciplinary approach that integrates fundamental lifestyle and dietary interventions, within a well-established therapeutic arsenal. Such an approach has been shown to effectively target the underlying metabolic disturbances, while also addressing organ-specific complications arising from these conditions. A multidisciplinary approach resulting from a broader vision of the MetS, which extends to the field of KSD, is important, as the clinical burden at the individual level and the economic consequences for health systems are obviously considerable. The management of dysmetabolic diseases therefore requires an expansion of the necessary holistic management that is already known towards the domain of KSD. It is also imperative to explicitly recognize the lithogenic component within the CKM syndrome. Local cell damage may occur quite early in the self-sustaining pathological process of lithogenesis, from the earlier stages of crystallization, even in the absence of structurally built stones. Integrating nephrolithiasis into the broader cardio-metabolic framework is therefore clinically relevant, not only for comprehensive patient care but also for targeted therapeutic interventions.
